# Estimating the impacts of recurrent and expanding coastal flooding on septic systems in Maryland’s Chesapeake Bay

**DOI:** 10.1007/s10584-026-04172-x

**Published:** 2026-04-07

**Authors:** Andre de Souza de Lima, Margaret A. Walls, Yanjun Liao, Emma DeAngeli, Allison Reilly, Nathan Boyd, Andrew Lazur, P. J. Ruess, Celso M. Ferreira

**Affiliations:** 1 Department of Civil, Environmental, and Infrastructure Engineering, George Mason University, Fairfax, VA USA; 2 Resources for the Future, Washington, DC USA; 3 Department of Civil and Environmental Engineering, Center for Risk and Reliability, University of Maryland, College Park, MD USA; 4 University of Maryland Extension, College Park, MD USA

**Keywords:** Onsite wastewater treatment systems, Sea-level rise, Lower eastern shore, ADCIRC+SWAN, Flood frequency

## Abstract

Projected sea-level rise (SLR) is expected to substantially expand coastal flood extents across Maryland’s Chesapeake Bay, with implications for decentralized wastewater infrastructure. This study integrates modeled coastal flooding scenarios from 2020 to 2060 with septic systems locations to quantify exposure under daily and annual flood conditions and identify areas where exposure intersects with high social vulnerability. Findings highlight spatial heterogeneity in flood impacts, with Dorchester County experiencing the largest flood extent and Worcester the steepest proportional increase in both flood extent and septic system exposure. By 2060, flood extent is projected to increase by 44% in Wicomico and exceed 260 km² in Somerset. Across the region, the number of affected septic systems more than doubles, with Worcester exhibiting a sevenfold increase from baseline and Dorchester surpassing 1,700 systems exposed. Flood frequency is also projected to increase, with hundreds of systems transitioning into monthly or weekly inundation by 2060, particularly in Dorchester and Somerset counties. Spatial aggregation identifies clusters of septic system exposure across the region. The spatial configuration of flood-exposed systems and social vulnerability varies across block groups, indicating that increasing flood exposure and recurrence affect communities with differing demographic characteristics.

**Supplementary Information:**

The online version contains supplementary material available at 10.1007/s10584-026-04172-x.

## Introduction

Flooding is among the most pressing natural hazards affecting coastal regions globally. It is increasingly exacerbated by climate-driven sea-level rise (SLR) and intensifying storm patterns, resulting in more frequent inundation and growing challenges for coastal communities (Kundzewicz et al. 2014; Kulp and Strauss 2019; Perks et al. 2023; Barnard et al. 2025). Particularly, chronic coastal (or nuisance) flooding, has become increasingly common in many low-lying areas, creating persistent disruptions that strain coastal infrastructure, public health systems, and local economies (Lane et al. 2013; Hernández-Delgado 2015). These dynamics also pose a substantial threat to Onsite Wastewater Treatment Systems (OWTS), especially septic systems^1^, which are notably vulnerable to flooding due to reliance on unsaturated soil conditions and sufficient vertical separation from groundwater tables to ensure effective wastewater treatment (Cox et al. 2019; Mitchell et al. 2021). As flood exposure increases in both intensity and persistence, the risks of septic system failure and groundwater contamination, and risks to public health are expected to rise. This is especially true in areas characterized by the co-occurrence of aging infrastructure and high social vulnerability (Koks et al. 2015; Rentschler et al. 2022; Maxcy-Brown et al. 2023; Gyimah et al. 2024), where limited adaptive capacity and pre-existing socioeconomic disadvantages can amplify exposure and reduce resilience to infrastructure failures.

Frequent and prolonged flooding can severely impair septic systems’ performance by limiting soil infiltration, causing backups, and elevating groundwater levels, which disrupt the treatment process and allow wastewater to reach the surface or nearby water bodies (Schaider et al. 2016; Sowah et al. 2017; McKenzie et al. 2021). Recurrent inundation, particularly when combined with sustained soil saturation, limits the soil dispersal component of a system or drain field’s ability to dry out between events, reducing recovery time and increasing the risk of hydraulic failure (Amer et al. 2023; Sprouse et al. 2024). While extreme flood events are often emphasized in research and planning, the cumulative impacts of chronic, lower-intensity flooding poses a substantial long-term threat to septic infrastructure (Kohler et al. 2020; Cantelon and Kurylyk 2024). Yet many infrastructure assessments still emphasize peak flood levels or isolated events, overlooking the repeated stressors that degrade system performance over time. Therefore, incorporating temporally explicit metrics is essential to better capture how recurring inundation can progressively undermine critical infrastructure, especially septic systems’ performance over time (Pezza and White 2021; Stempel et al. 2021).

The consequences of recurring septic system failures are far-reaching, as flooding becomes more frequent, repeated disruptions can compromise essential sanitation functions, resulting in reduced habitability, costly system repairs, and increased reliance on emergency response mechanisms. These disruptions strain not only individual households, but also expose systemic gaps in infrastructure resilience, especially in unsewered, rural, or historically underinvested areas where septic systems are the sole means of wastewater management (Borges Pedro et al. 2020; Wells et al. 2022). Moreover, these risks are often magnified in underbounded and unincorporated communities, which often face long-standing service inequities tied to fragmented governance, limited access to public utilities, and the absence of regulatory enforcement (Capps et al. 2020; Allaire et al. 2024). In this broader context, septic system vulnerability reflects more than the effects of environmental stressors alone, it demonstrates how infrastructure condition, institutional support, and patterns of land use interact to shape community-level risk. While chronic flooding accelerates system degradation, the magnitude and persistence of its impacts often depend on where failures occur and who is most affected. These recurring stresses can produce cascading effects, thus disrupting household functioning, limiting access to basic services, and amplifying vulnerabilities in already at-risk populations (Malloy and Ashcraft 2020; Capps et al. 2021).

Social vulnerability is a critical lens for understanding how social structures and disparities shape the distribution and consequences of environmental hazards across population groups (Cutter et al. 2003; Madajewicz 2020; Hendricks and Van Zandt 2021). These conditions influence not only baseline risk but also access to adaptation resources and participation in institutional decision-making (Adger 2003; Eriksen et al. 2015; Shi et al. 2016). Social Vulnerability Indices (SVIs) operationalize these factors, facilitating spatial assessments that identify groups with constrained adaptive capacity (Tate 2013; Nguyen et al. 2017; Tanir et al. 2021). Across the United States and globally, socially vulnerable populations tend to cluster in areas with elevated hazard exposure, compounding risks in ways that are not solely explained by geography (Rentschler et al. 2022). Regarding flooding, this intersection produces measurable disparities, and marginalized groups are more likely to reside in low-elevation or poorly drained areas and less likely to receive protective infrastructure investments or timely post-disaster support (Collins et al. 2018; Tate et al. 2021). These vulnerabilities are further magnified where decentralized wastewater systems are relied upon (Leker and MacDonald Gibson 2018). Communities without access to centralized sanitation, especially in unincorporated, rural, or historically underserved regions, must depend on septic systems that require regular maintenance and are particularly sensitive to inundation (Mitchell et al. 2021; Kryston et al. 2024; Sprouse et al. 2024). Under these conditions, the convergence of chronic flooding, infrastructural fragility, and systemic neglect leads to recurring sanitation failures with public health consequences, disproportionately burdening those with the fewest resources to respond.

In this context, this study investigates the vulnerability of OWTS (i.e., septic systems) to long-term coastal flooding in the Lower Eastern Shore region, located within Maryland’s Chesapeake Bay (Supplementary Material 1). Hydrodynamic and wave modeling was employed to identify flood occurrence across a representative year (2020), with this framework applied to projected future sea-level conditions at decadal intervals (2030-2060) to evaluate shifts in coastal inundation patterns over time. Resulting flood extents are then overlaid with septic system locations to quantify exposure frequency and identify infrastructure subject to recurrent inundation under projected future conditions. These results are subsequently integrated with an SVI to assess where projected flood exposure coincides with patterns of socioeconomic characteristics. This analysis enables a spatially explicit understanding of how climate-driven changes in coastal flooding may interact with infrastructural and demographic conditions to amplify environmental vulnerability across Maryland’s Chesapeake Bay.

## Methodology

This study employs an integrated numerical modeling and spatial analysis framework (Supplementary Material 2) to evaluate the long-term vulnerability of septic systems to coastal flooding and projected SLR in Maryland’s Lower Eastern Shore. The analysis focuses on Dorchester, Wicomico, Somerset, and Worcester counties, which, along with the greater Chesapeake Bay region, experience some of the highest relative SLR rates along the U.S. Atlantic coast. This is driven by the combination of rising coastal water levels and pronounced regional land subsidence (Ezer 2023). The region’s extensive low-lying areas, and limited drainage capacity compound flood susceptibility, while socioeconomic conditions, marked by some of the lowest income levels in the state, contribute to high social vulnerability (Martin et al. 2022). Moreover, this is also a region where the majority of households rely on septic systems for wastewater treatment, thus elevating the infrastructural risks posed by recurrent flooding (Supplementary Material 1). Total water levels were simulated using the coupled ADCIRC+SWAN model over a full annual period (i.e., 2020). Spatial downscaling was applied to model outputs to generate high-resolution maximum flood extents for each day, allowing identification of the number of days under flooding conditions – a key metric for estimating septic system exposure to environmental hazards. Septic system locations were estimated through a spatial association method that linked primary structures (i.e., building footprints) to parcels, excluding areas served by centralized sewer infrastructure. These estimated locations were intersected with daily flood extents to quantify the frequency and spatial distribution of inundation under both present and projected SLR scenarios. Flood projections were developed for multiple decadal SLR intervals (i.e., 2030, 2040, 2050, and 2060) to assess changes in exposure patterns over time. Finally, results were integrated with a regional SVI to evaluate the co-occurrence of physical exposure and underlying social vulnerability.

### Modeling framework and setup

The simulation of coastal water levels in this study was conducted using the coupled ADCIRC+SWAN model, which integrates a two-dimensional, depth-integrated hydrodynamic model with a third-generation spectral wave model to represent coastal processes under both normal and storm surge conditions. The ADvanced CIRCulation model (ADCIRC) computes water levels by solving the Generalized Wave Continuity Equation (GWCE) and horizontal depth-averaged currents using the vertically integrated shallow water momentum equations (Luettich et al. 1992). Implemented over an unstructured triangular mesh, ADCIRC incorporates physical processes such as wind stress, atmospheric pressure gradients, bottom friction, Coriolis forcing, and nonlinear advection. The use of an unstructured grid enables spatial resolution to be refined in nearshore and estuarine regions, allowing the model to accurately represent inundation across complex coastal geometries. ADCIRC has been extensively applied in coastal hydrodynamic studies worldwide, including simulations of astronomical tides, storm surge, and total water levels associated with flooding events (Sebastian et al. 2014; Wang et al. 2018; Deb and Ferreira 2018; de Lima et al. 2020). The Simulating WAves Nearshore (SWAN) model is a phase-averaged spectral wave model that solves the wave action balance equation in Eulerian form (Booij et al. 1999). The model simulates the generation and evolution of wave energy through wind input, white capping, quadruplet wave–wave interactions, bottom friction, and depth-induced breaking. Its formulation allows wave propagation to be resolved across spatially variable bathymetry and topography. While wave fields were not explicitly analyzed in this study, SWAN was coupled with ADCIRC to account for wave-induced setup, which contributes to total water levels, particularly during storm events in low-lying coastal areas.

When coupled, ADCIRC+SWAN (Dietrich et al. 2011) exchange hydrodynamic and wave information at each time step through a two-way feedback approach. SWAN first accesses wind speed, water levels, and current velocities provided by ADCIRC to compute the wave field and associated radiation stress gradients. These gradients are then passed back to ADCIRC, where they modify the momentum equations and contribute to updated water surface elevations and currents. This two-way interaction ensures that the feedback between surge and wave processes are dynamically captured, improving the accuracy of total water level estimates under both normal and storm surge conditions. Groundwater table dynamics were not explicitly simulated, as the analysis was limited to coastal flooding and SLR–driven surface inundation processes. The numerical framework employed in this study is based on the model developed, calibrated and validated by Khalid and Ferreira (2020) for the Chesapeake Bay and adjacent Atlantic coast, which has also been operationalized within a real-time forecasting framework. The model was forced with hourly winds at 10 m above the sea surface and mean sea level pressure from the European Centre for Medium-Range Weather Forecasts Reanalysis v5 (ERA5) (Hersbach et al. 2020), and eight primary tidal constituents derived from TPXO (Egbert and Erofeeva 2002), (i.e., K1, K2, O1, M2, N2, P1, Q1, and S2), whereas the high-resolution numerical mesh is based on regional topobathymetric data from the Coastal National Elevation Database - Topobathymetric Digital Elevation Model (Danielson et al. 2018). Relative SLR was incorporated into the model framework by adjusting baseline water levels according to projections for the Cambridge (MD) tide gauge, as reported in the 2023 Maryland Sea-Level Rise Projections (Boesch et al. 2023). These projections are based on probabilistic assessments derived from IPCC Sixth Assessment Report (AR6) global mean sea-level estimates, combined with regional adjustments for land subsidence. Median SLR values associated with the SSP2-4.5 emissions scenario were applied at decadal intervals from 2030 to 2060 (Supplementary Material 3).

Flood extent was used as the exposure metric to support scalable, spatially resolved assessment of parcel-level inundation across daily simulations representing baseline (i.e., 2020) and projected SLR conditions for 2030, 2040, 2050, and 2060. This metric captures the physical footprint of coastal flooding in a way that is directly relevant to septic system function. Inundation zones represent hydrologically meaningful conditions, as the presence of flooding can reflect elevated groundwater, saturated soils, and recurring exposure that may compromise system performance over time (Amer et al. 2023). The year 2020 was selected as the baseline scenario because it exhibited a limited number of coastal flooding events across the study area. Although a number of minor and moderate tidal flood events were recorded by NOAA tide gauges, no extreme events occurred during the year in Maryland’s Lower Eastern Shore. This provided a conservative reference period to evaluate the influence of SLR on recurrent, low-magnitude flooding in the absence of major storm-driven events. Flood extents were derived from ADCIRC+SWAN model outputs and downscaled using Kalpana, a Python module to convert hydrodynamic model outputs to geospatial vector formats (Rucker et al. 2021). For each simulation day, maximum water surface elevations were extracted at all numerical mesh nodes, and flood extents were extrapolated onto a 30 m elevation grid derived from the Coastal National Elevation Database DEM. Inundated areas were delineated where modeled water levels exceeded terrain elevation, with hydraulic connectivity constraints applied to restrict lateral spread in low-relief terrain. The resulting sequence of daily flood maps was used for parcel-scale exposure analysis, with spatial analysis and map visualization conducted in ArcGIS Pro (version 3.5) and plots generated in Python (version 3.11).

### Septic exposure analysis

Annual maximum flood extent was defined as the union of all areas inundated at least once during a given year, representing the cumulative spatial footprint of flooding rather than a single event. These annual maximum flood layers were intersected with septic system locations to identify systems projected to experience at least one day of inundation per year under each scenario. Flood frequency was quantified by calculating the number of inundation days per year for each system based on daily modeled flood extents, and systems were classified into recurrence categories of at least once per year, once every six months, once per month, once per week, or no flooding. Septic system locations were inferred from the Maryland’s Parcel Points and Polygons geodatabase (Maryland Department of Planning 2022), which includes an attribute denoting sewer connection status. To account for missing data and potential inaccuracies in infrastructure delineation, a spatial classification that integrates parcel-level attributes with generalized sewer service boundaries was applied (Maryland Department of Planning 2024), assuming parcels located outside documented service areas are served by septic systems. For each identified parcel, the likely location of the septic system was approximated using a 15 m search radius around the primary residential structure to capture the typical placement of both the septic tank and drainage field. This spatial assumption reflects standard siting practices and regulatory setbacks in the state of Maryland. This additional step allows for a more accurate assessment of flooding of the septic drain field, rather than other areas of the parcel.

To characterize social vulnerability, we used indicators from the U.S. Environmental Protection Agency’s EJScreen dataset (version 2.3) (EPA, 2024a), which provides nationally standardized measures of demographic and environmental risk at the Census block group level. Specifically, we incorporated the Demographic Index (DI) and the Supplemental Demographic Index (SDI), both of which are constructed as unweighted averages of Z-scores derived from selected variables standardized to national distributions. For both indices, Z-scores for each component variable are calculated using the national mean and standard deviation, thereby capturing how each block group deviates from national demographic norms. These standardized values are then averaged and converted to percentiles to facilitate comparability across regions. The DI reflects the average of Z-scores for the percent of the population classified as low-income and the percent identified as people of color. The SDI extends this characterization by incorporating five additional indicators: percent of the population with limited English proficiency, less than high school education, with a disability, experiencing low life expectancy, and also low income (EPA, 2024b). While both indices are constructed using similar methods, a key distinction is that the DI explicitly incorporates race (percent people of color), whereas the SDI does not. This supports treating them as complementary measures to capture different dimensions of demographic vulnerability in the study area. Finally, parcel-level flood exposure results were summarized at the block group level to align with the spatial resolution of the vulnerability data. This enabled comparison of inundation frequency with demographic patterns and identification of block groups where both exposure and vulnerability were elevated. EJScreen additionally includes many unique environmental justice indices which combine the DI and SDI with environmental burden indices also included in the EJScreen portal. While these offer value for broader environmental assessments, this study and its focus on flooding did not fit well with any of these EJ indicators. Consequently, flood results were related directly to the socioeconomic indicators, DI and SDI, rather than to environmental justice indices.

## Results

### Changes in flood extent and duration over time

**Fig. 1:**
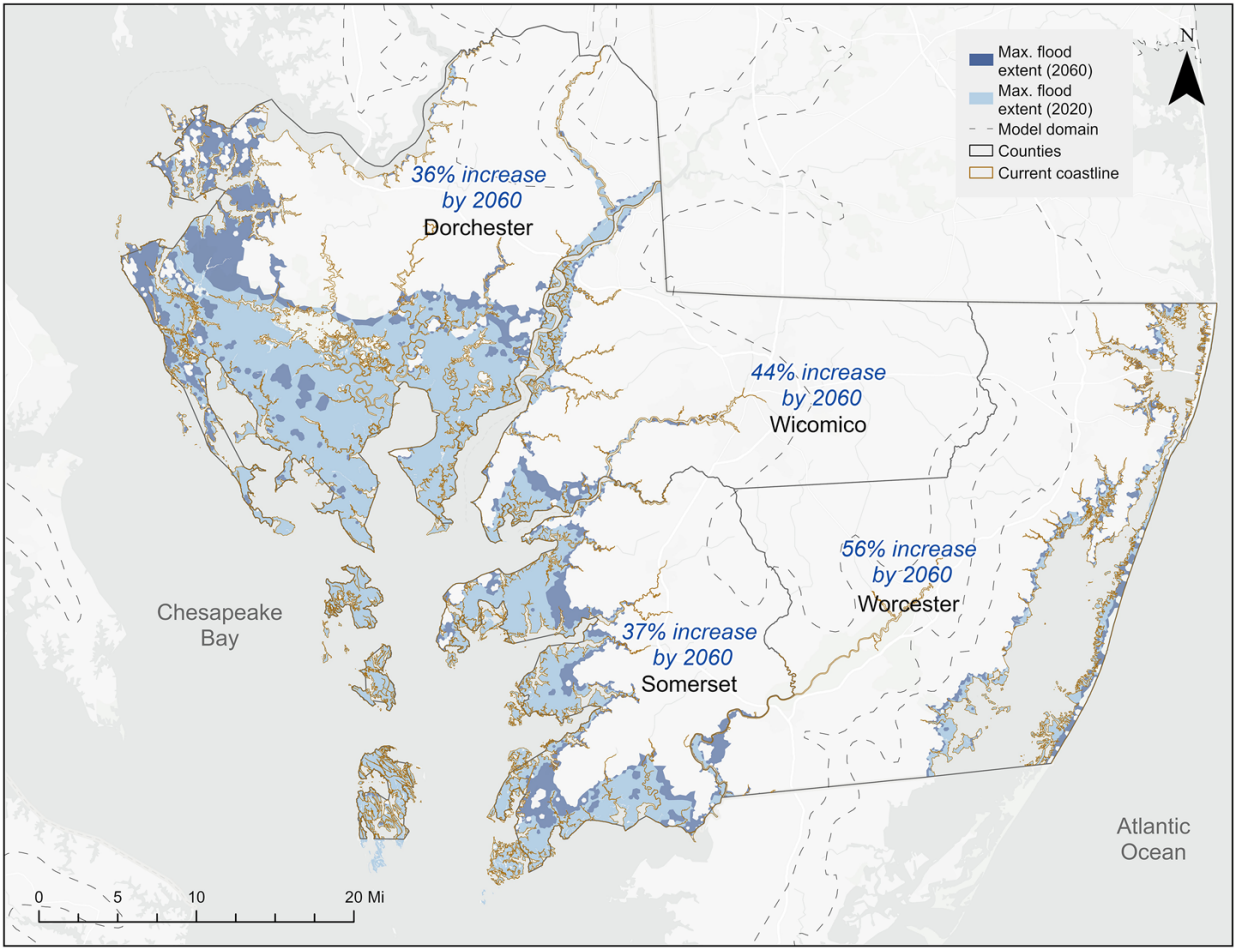
Modeled maximum flood extents for Maryland’s Eastern Shore under current (2020) and projected future (2060) sea level conditions. Shaded areas represent zones estimated to experience at least one coastal flooding event per year under each scenario

Flood exposure was evaluated using modeled daily and annual inundation extents as defined in Sect. 2.2. Under current conditions, flooding is already impacting Maryland’s Eastern Shore. Our modeled maximum flood extent in 2020 covers approximately 18% of the study region’s land area, with this coverage projected to increase to 24% by 2060 (Fig. 1). County-level relative increases in flood extent over this period range from 35.7% in Dorchester to 56.5% in Worcester. Worcester exhibits the highest proportional increase, while Dorchester, already the most extensively affected today, maintains the largest absolute flood inundation area. Wicomico also experiences a notable relative increase (43.7%) despite its smaller baseline extent (Supplementary Material 4). By 2060, modeled flood extent represents approximately 43% of Dorchester’s land area, 33% of Somerset’s, 27% of Wicomico’s, and 9% of Worcester’s. These values indicate that Dorchester remains the most heavily affected, both in absolute and relative terms. Flood extent expands in approximate decadal increments, corresponding with modeled SLR projections of 0.19 m by 2030, 0.28 m by 2040, 0.38 m by 2050, and 0.47 m by 2060 relative to the 2005 baseline (Supplementary Material 3). These SLR increments result in cumulative increases in flood area of 147 km² (2030), 202 km² (2040), 255 km² (2050), and 290 km² (2060), relative to the 2020 baseline for the entire region (Supplementary Material 4).

As shown in Supplementary Material 5, the number of septic systems within the maximum flood extent increases in all counties across the modeled period. Somerset experiences the largest relative increase, from 255 impacted systems in 2020 to 1,125 by 2060 (+341%), which corresponds to 17.2% of all systems in the county, the highest proportion observed. Notably, exposure in Somerset begins to rise sharply between 2020 and 2030, with the number of affected systems more than doubling in that interval. Dorchester sees a 133.8% increase in impacted systems (from 731 to 1,709), representing 15.4% of its total. The largest single-decade increase in absolute numbers for Dorchester occurs between 2030 and 2040 (+191 systems). Worcester increases from 43 to 346 systems (+704.7%), while Wicomico rises from 39 to 95 systems (+143.6%), representing 1.8% and 0.4% of their totals, respectively. The largest increases for Worcester and Somerset both occur between 2030 and 2040.

**Fig. 2:**
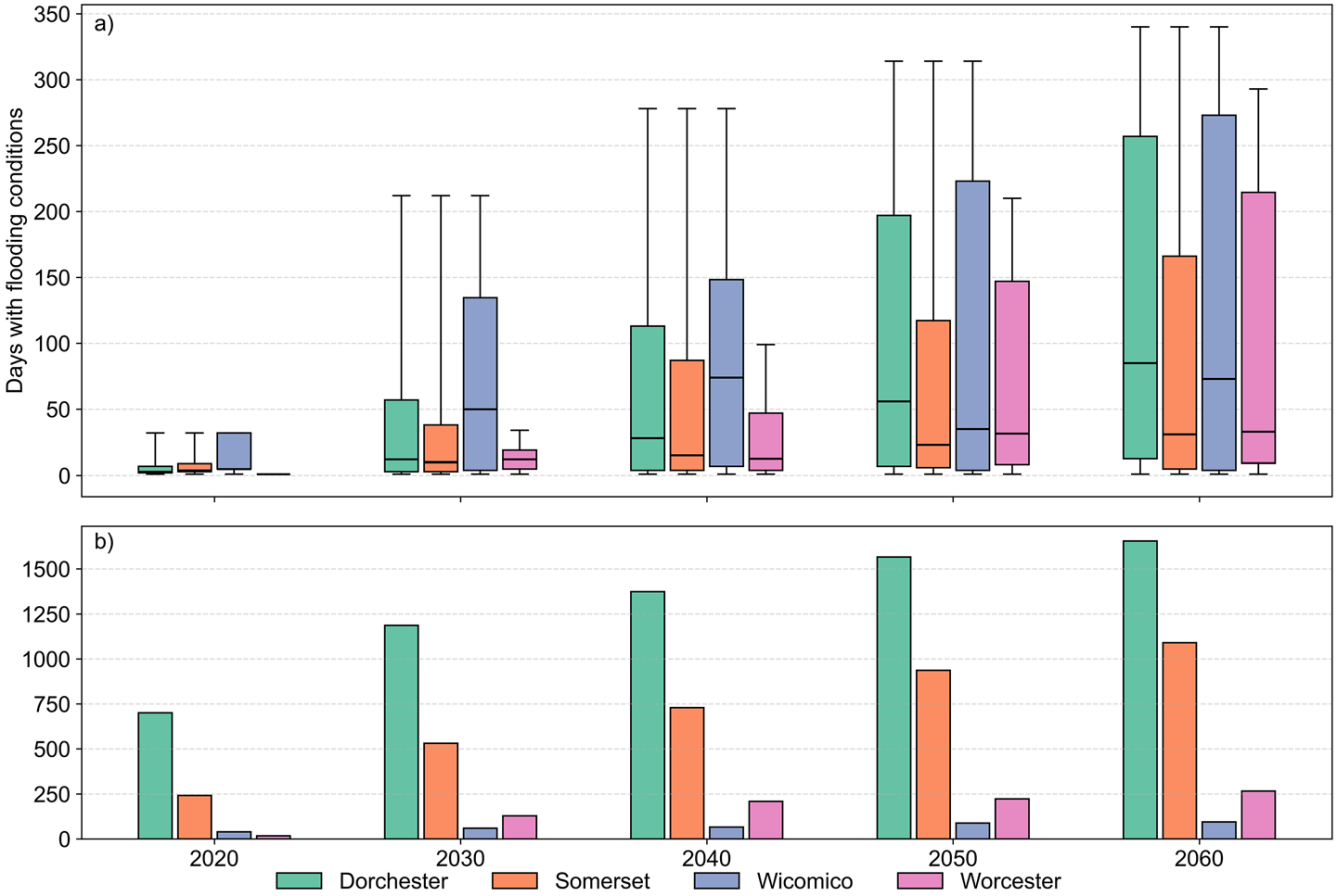
(**a**) Distribution of annual flood-day counts among flooded septic systems from 2020 to 2060 by county. (**b**) Number of septic systems projected to experience at least one day of flooding per year under baseline and SLR scenarios

In addition to annual extent, flood frequency was assessed using daily flood layers. Across all counties, the number of flood days per year increases substantially over time. In Dorchester, the average rises from 5 days in 2020 to 128 days in 2060, with some systems reaching up to 340 days. Wicomico follows a similar trajectory, increasing from 14 to 134 days. Somerset increases from 7 to 92, while Worcester shows the steepest proportional growth, rising from 1 to 100 days, with a maximum of 293 days in 2060. Figure 2 summarizes both the number of potentially impacted septic systems and their exposure frequency over time. Bar plots show the total count of affected systems by county for each modeled year, while overlaid markers represent the corresponding average and maximum flood days. Although Worcester and Wicomico have fewer impacted systems overall, the flood frequency among those affected systems is comparable to Dorchester and Somerset, with similar average and maximum values observed by 2060.

**Fig. 3:**
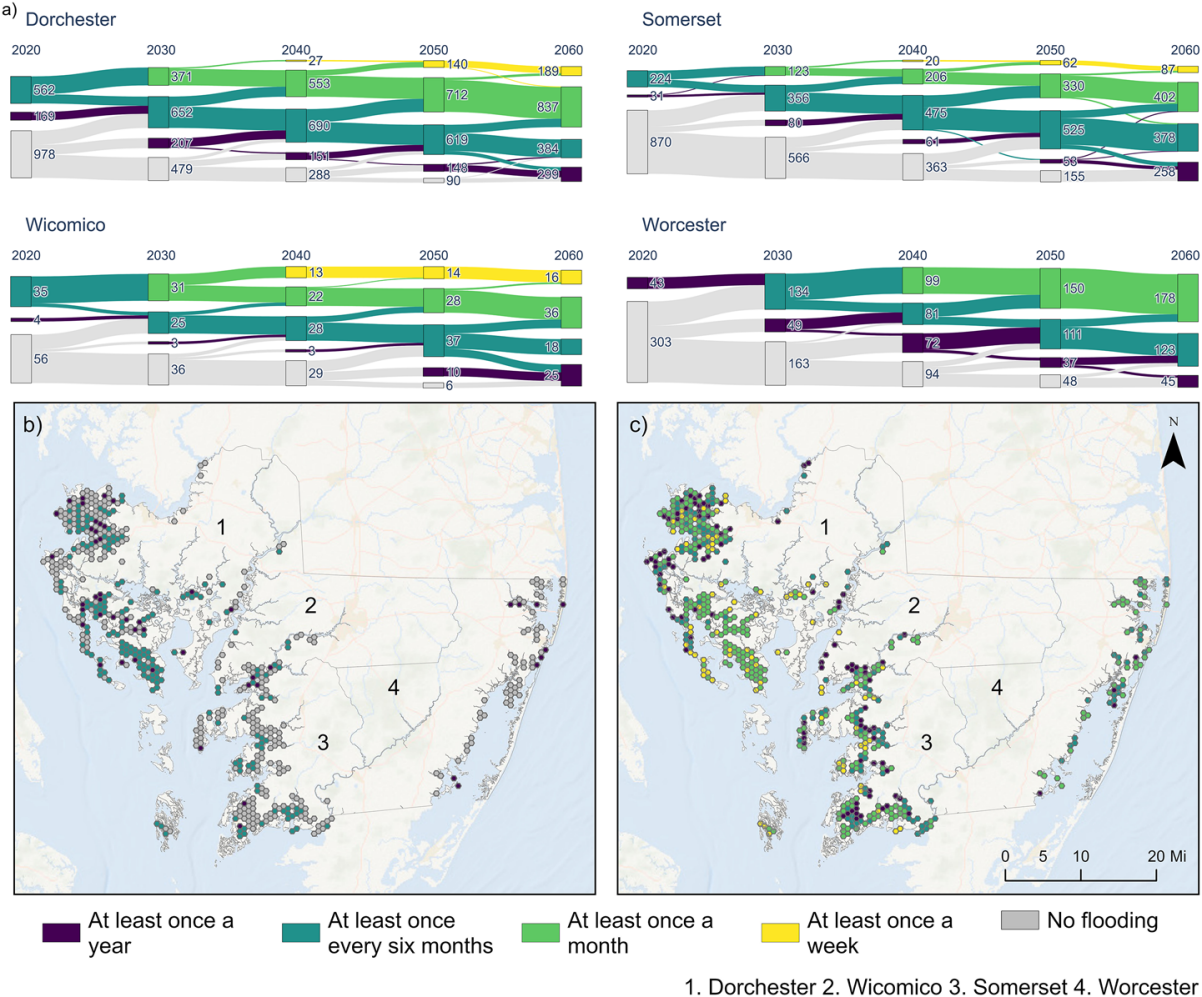
(**a**) Transitions in flood frequency exposure for septic systems from 2020 to 2060 under projected SLR. Systems are grouped by recurrence categories based on the number of inundation days per year; (**b**-**c**) Spatial distribution of dominant flood frequency classes for septic systems in 2020 (**b**) and 2060 (**c**), aggregated by 1 km² hexagon cells. Each hexagon reflects the most prevalent flood frequency class within its area; this representation does not imply uniform conditions across the entire cell

Figure 3 illustrates the transitions between categories from 2020 to 2060 under projected SLR. For example, in Dorchester County, 978 systems were never flooded in 2020, but by 2030 more than half of these had moved into one of the flooded categories, leaving only 479 never flooded. Exposure intensifies over the future decades. None of the four counties had septic systems that experienced flooding more than once a month in 2020, but three of the four counties had some systems in this category by 2030 and all four counties by 2040. In Dorchester County, nearly 190 systems experienced flooding as frequently as once per week in 2060, the most of any county. Somerset County showed similar trends, with exposure increasing over time. Of the systems projected to be impacted by flooding by 2060, most were not yet exposed in 2020. By 2050, however, more than 900 systems had entered one of the flood recurrence categories, and by 2060, 87 of these can potentially experience flooding on a weekly basis. Wicomico County stands out with a growing share of its septic systems flooding at least once per week; by 2060, 17% of the systems in the county are projected to flood at least once a week. Worcester County was the only jurisdiction in which no systems reached weekly flooding by 2060. Nonetheless, the number of exposed systems increased substantially over time. In 2020, only 43 systems flooded annually; by 2060, 346 systems flooded at least once a year, including 178 systems experiencing monthly flooding. A complementary analysis of the maximum number of consecutive flooded days per system, used to evaluate persistence and uninterrupted inundation, is provided in Supplementary Material 7.

To examine the spatial progression of flood recurrence, septic system exposure was summarized using a 1 km² hexagonal tessellation across the study area (Fig. 3). Each hexagon was assigned the modal flood frequency class based on the systems located within it, representing the most commonly occurring recurrence level per time step (i.e., 2020 and 2060; Fig. 3b and c). Thus, the maps illustrate changes in the predominant flood frequency across space, while not implying uniform conditions within each hexagon. In 2020 (Fig. 3b), the dominant flood frequency in most hexagon cells corresponded to either no flooding or low-frequency exposure (annual or biannual), with the highest concentrations of affected areas observed in Dorchester, Somerset, and to a lesser extent, Worcester counties. Dorchester exhibited the most extensive early exposure, with a large share of cells already reflecting recurring inundation, primarily in the southern portion of the county. Somerset and Wicomico showed smaller but spatially coherent clusters of affected bins, while Worcester remained largely unexposed. Hexagons classified as “no flooding” in 2020, but projected to transition into flood-affected classes by 2060, were most numerous in Dorchester (139 cells), followed by Somerset (122), Worcester (53), and Wicomico (23). By 2060 (Fig. 3c), higher-frequency flood classes became more prominent across the region. In Dorchester, 142 hexagon cells were dominated by monthly recurrence and 51 by weekly flooding. Somerset followed with 68 monthly and 21 weekly cells, indicating a substantial shift toward more frequent inundation. Wicomico also showed increases, with 14 monthly and 4 weekly cells by 2060, despite having minimal exposure in 2020. Worcester remained the only county without any cells dominated by weekly flooding, although 33 cells reached monthly recurrence (Supplementary Material 6).

### Socioeconomic groups and exposure

**Fig. 4:**
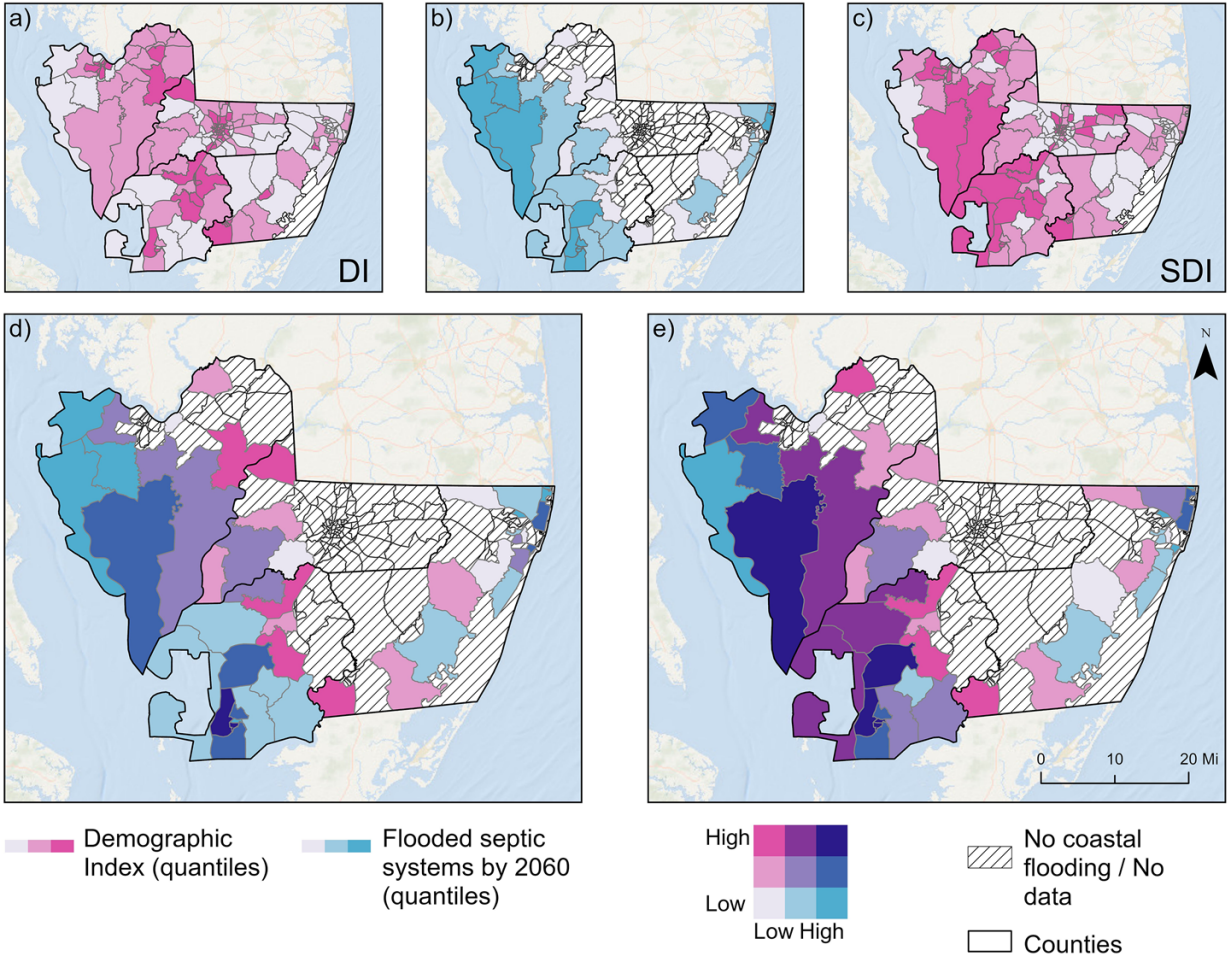
Spatial distribution of social vulnerability and projected flood exposure. **a**) Demographic Index (DI); **b**) projected flooded septic systems by 2060 normalized by the existing total number of septic systems within each Census block group; **c**) Supplemental Demographic Index (SDI); **d**) bivariate map of DI and projected flooding exposure; **e**) bivariate map of SDI and projected flooding exposure. Darker purple tones represent Census block groups with high values in both vulnerability and projected flood exposure

Socioeconomic conditions are an important dimension of flood-related impacts, as certain populations may have fewer resources to prevent, respond to, or recover from damage. To evaluate the distribution of social vulnerability across the study area, we used the Demographic Index (DI), based on percent low income and percent people of color, and the Supplemental Demographic Index (SDI), based on percent low life expectancy, low income, with a disability, limited English proficiency, and less than high school education from the EPA’s EJScreen dataset (EPA, 2024a). Both indices are normalized measures in which higher values indicate greater relative social vulnerability within the region, whereas lower values reflect comparatively lower vulnerability. In Maryland’s Lower Eastern Shore DI values ranged from near 0.2 in the North of Worcester County to above 3.3 in central urban areas of Dorchester, Somerset, and Wicomico, indicating substantial variability in demographic vulnerability across the region at Census block group level (Fig. 4a). Compared to the four-county regional average of 1.32, Worcester’s mean DI (0.94) was notably lower, while all other counties had average values above the regional mean. SDI patterns were similarly uneven (Fig. 4b). Maximum SDI values exceeded 3.0 in Wicomico (3.74) and Dorchester (3.08). Wicomico had the highest individual SDI value, while Somerset had the highest mean (1.80), suggesting a broader prevalence of elevated social vulnerability in that county. Moreover, all counties except Worcester had block groups above the regional average of 1.62. In contrast, minimum SDI values were below 1.0 in northern Worcester and central Wicomico, suggesting localized areas of relatively low vulnerability.

In 2020, flooded septic systems were located in 35 Census block groups distributed across all four counties. These block groups varied substantially in both the number of affected parcels and their demographic profiles, as characterized by their respective DI and SDI. The number of flooded parcels per block group ranged from 1 to over 300, with the largest clusters concentrated in Dorchester and Somerset counties. Among these exposed areas, DI values ranged from 0.4 to 2.25 (mean: 1.04), and SDI values from 0.93 to 2.38 (mean: 1.57). Compared to the four-county regional averages (DI: 1.32; SDI: 1.58), these values suggest that many of the block groups affected in 2020 fell near or slightly below the regional mean for DI, while SDI values showed greater variability, ranging widely around the mean. Most block groups exhibited moderate values on both indices, but a subset of high-impact areas, particularly in Somerset, combined relatively high DI and SDI values with large numbers of flooded septic systems. Dorchester also had multiple Census block groups with elevated SDI and moderate DI, while Worcester’s affected areas were concentrated in block groups with relatively low DI and SDI. Wicomico had fewer affected block groups overall, but some were located in demographically diverse areas (Supplementary Material 8).

Block groups with increased septic system exposure between 2020 and 2060 were identified across all four counties, with notable variation in both the magnitude of change and the associated demographic characteristics (Supplementary Material 9). In total, 46 block groups exhibited a net increase in the number of impacted systems, reflecting the expansion of flood-prone areas due to SLR. These groups included locations that were unaffected in 2020 but became exposed by 2060, as well as those already experiencing flooding where exposure intensified. The largest absolute increases in impacted systems occurred in Dorchester and Somerset counties, with individual block groups showing increases of over 400 and 200 septic systems, respectively. Several of these block groups already had substantial exposure in 2020, but the number of affected systems more than tripled by 2060. In Dorchester, multiple block groups with high increases had relatively low DI (<1.0) and SDI scores (<1.4), while in Somerset, the increase occurred across a broader vulnerability range, including block groups with both high DI (>2.0) and high SDI (>2.2). However, these highest vulnerability block groups were not among those with the largest increases in impacted systems. Wicomico and Worcester showed less substantial increases, averaging 11 and 17 additional impacted systems per block group, respectively. Only one block group in Worcester is projected to experience an increase exceeding 100 septic systems. Nonetheless, several affected areas in both counties exhibit SDI values at or above the regional mean, particularly in Worcester, while DI values in these counties remain generally below the regional average.

Finally, the relationship between projected flood exposure and demographic vulnerability was examined by comparing 2060 septic system impacts with DI and SDI values across Census block groups (Fig. 4). All variables were classified into three quantile-based categories for mapping and exposure was normalized by the total number of septic systems in each block group. DI values (Fig. 4a) were particularly highest in central and southern Somerset and in central Wicomico and Dorchester. Normalized flood exposure (Fig. 4b) showed the greatest concentrations in Dorchester and Somerset. SDI values (Fig. 4c) were elevated across much of Somerset and Dorchester, particularly in nearshore areas, and in central Wicomico. High values were more dispersed compared to DI, though in Worcester only one inland block group showed elevated SDI. In the bivariate map combining DI and projected exposure (Fig. 4d), only one block group in southern Somerset falls into the highest class for both variables. Two adjacent block groups in the same area also show the highest flood exposure combined with moderate DI values. A similar pattern is observed in Dorchester, where several block groups have high exposure but only moderate levels of demographic vulnerability. When combining SDI and exposure (Fig. 4e), two nearshore block groups in Somerset fall into the highest category for both variables. An additional three block groups in the county exhibit the highest SDI values but only moderate levels of flood exposure. A similar pattern is observed in Dorchester, where a few block groups also show high vulnerability combined with medium exposure levels. In contrast, patterns in Wicomico and Worcester remain consistent with those observed in the DI-based map, where the most vulnerable block groups are located further inland and do not overlap with projected flood exposure.

## Discussion

Projected SLR results in a steady expansion of annual flood extent across Maryland’s Lower Eastern Shore, with county specific patterns reflecting differences in topography, coastal morphology, and existing exposure (Wu et al. 2009; Cassalho et al. 2023; de Lima et al. 2024). Dorchester County remains the most extensively affected throughout the modeled period, with inundated land area increasing by approximately 36% from 2020 to 2060, consistent with prior findings that identify Dorchester as highly vulnerable due to rapid relative SLR, marsh loss, and low relief (Taylor et al. 2020; Li et al. 2020; Jasour et al. 2022). Somerset and Worcester can also experience notable growth in flooded area, reaching 270 km² and 104 km², respectively, by mid-century, aligning with regional SLR projections that amplify surge-driven flooding and expand flood extent. While Wicomico covers a smaller absolute area, it registers a 44% potential increase relative to its 2020 baseline, indicating that even less exposed areas may face escalating risk as non-inundated zones experience greater episodic flooding and associated damages. The varying rates of flood extent expansion across jurisdictions reflect localized landscape features and elevation gradients, which in turn shape both community and ecosystem vulnerability. Overall, the progressive increase in inundated land area across all four counties points to a broadening geographic footprint of coastal flooding with implications for land use, infrastructure planning, and long-term risk exposure in the region (Michael 2007; Rezaie et al. 2021; Martin et al. 2022).

The spatial intersection of modeled flood extents with septic system locations reveals a clear trajectory of increasing exposure under projected SLR scenarios. By 2060, all four counties exhibit marked increases in the number of septic systems situated within areas projected to flood annually, with Somerset and Dorchester accounting for the largest totals. While Dorchester maintains the highest absolute number of exposed systems, the steepest proportional growth occurs in Worcester, where the number of systems exposed to at least annual flooding grows more than sevenfold. These spatial trends correspond with known failure mechanisms for OWTS, which are vulnerable not only to surface inundation but also to rising groundwater levels, both of which can increase under SLR conditions (Cox et al. 2019; Kohler et al. 2020; Cantelon and Kurylyk 2024). Beyond annual exposure, projected flood frequency increases significantly across affected systems. In Dorchester, where more than 1,700 systems are projected to be exposed by 2060, over 45% face at least monthly flooding, with nearly 12% experiencing weekly inundation. Somerset county, with 1,125 impacted systems projected to be impacted by 2060, follows closely, since up to 35% of its total could face monthly flooding, whereas 8% can reach weekly flooding. In Worcester, 51% of affected systems are projected to flood monthly, although none reach weekly recurrence. Wicomico, despite its smaller scale, shows an upward trend in recurrence, with weekly flooding emerging by 2040. These shifts indicate that a substantial subset of exposed systems may face chronic hydrologic stress, since frequent saturation undermines septic function, especially where shallow groundwater or limited drainage reduces unsaturated zone depth, and may trigger effluent surfacing or hydraulic failure over time (Pezza and White 2021; Hoghooghi et al. 2021; Bosserelle et al. 2022; O’Driscoll et al. 2024; Sprouse et al. 2024).

The findings from Maryland’s Lower Eastern Shore contribute to a growing body of literature documenting the increasing vulnerability of OWTS under projected SLR, rising groundwater, and chronic inundation. The documented transition from episodic to frequent or near-continuous flooding mirrors concerns raised by Cox et al. (2019), who provided early evidence that rising groundwater tables linked to SLR pose a growing threat to septic system functionality, especially in low-lying coastal areas, which are also exposed to storm surge (Cox et al. 2020). This mechanism is further substantiated by O’Driscoll et al. (2024), who linked elevated water tables with compromised vertical separation distance, a key factor in OWTS treatment performance. The observed increase in flood recurrence aligns with assessments by Sprouse et al. (2024) and Hoghooghi et al. (2021), which emphasize that chronic hydrologic exposure exacerbates treatment failures by sustaining saturated conditions around drain fields. Notably, Pezza and White (2021) documented how the duration of flooding directly influences infrastructure degradation, a dynamic particularly relevant to the patterns of monthly and weekly flooding emerging in this study. Bosserelle et al. (2022) further demonstrated the compounded effects of SLR and shallow groundwater on wastewater exposure risk, reinforcing the interpretation that even moderate flood depths, if frequent or prolonged, can produce substantial system stress. Furthermore, while Kohler et al. (2020) and Cantelon and Kurtlyk (2024) document hydrologic drivers relevant to septic system exposure (i.e., soil saturation from extreme rainfall and subsurface flooding from coastal forcing) no prior studies to our knowledge have incorporated flood recurrence and daily inundation metrics into septic vulnerability assessment, a gap being addressed by this study. At the same time, systematic documentation of septic system failures attributable specifically to coastal flooding remains limited in the study region, highlighting the need for future work that integrates modeled exposure with observed system performance and reported impacts.

The growing evidence on septic system problems in areas subject to SLR suggests a need for changes to state septic system policies and regulations. States set a variety of specific regulations for siting of new systems, including minimum depth-to-groundwater requirements, minimum distances to water bodies, and some limitations on systems in floodplains but, with the exception of Virginia, no state along the Atlantic or Gulf Coast takes future climate conditions into account. With a 30-year lifespan for a typical septic drain field, considering future conditions when siting a new system can be critical. Addressing problems with current systems requires better identification of where those systems are, information that most states are not routinely collecting. Finally, public funding or private financing will be needed to address the problem through selective system upgrades, sewer expansion, and in the most problematic areas, property buyouts and household relocations. In smaller communities with more limited resources, public funding will be especially critical. At present, funding along these lines is limited. Most funding for water infrastructure investments in the United States goes toward upgrades to wastewater treatment plants; onsite systems receive far less attention. Maryland is one of the few states that has its own public funding program for wastewater improvements. The Bay Restoration Fund uses revenues from a tax on Maryland households in the Chesapeake Bay watershed for wastewater treatment plant upgrades, septic system replacements, and other investments to improve Bay water quality, but the program spends only about 10% of its funds each year on septic (DeAngeli et al. 2026).

While the broader spatial convergence of increased septic system flood exposure and uneven social vulnerability across Maryland’s Lower Eastern Shore reflects patterns documented in environmental justice and flood risk research, detailed analysis reveals that the most flood-exposed areas do not consistently coincide with the highest social vulnerability indices. This aligns with prior findings that hazard exposure and social vulnerability may diverge at finer spatial scales (Koks et al. 2015; Collins et al. 2018; Tate et al. 2021). The presence of demographically vulnerable block groups experiencing intensified exposure over time highlights the relevance of integrated assessments combining flood exposure and vulnerability, particularly for decentralized infrastructure often omitted from centralized resilience strategies (Tanir et al. 2024; Allaire et al. 2024). In the same context, regional assessments similarly find that rural and lower-income areas reliant on onsite wastewater systems are disproportionately at risk under future flooding scenarios (Sprouse et al. 2024). These findings point to the need for analytic frameworks that can capture both exposure magnitude and relative vulnerability across diverse spatial and demographic contexts. The use of normalized exposure metrics and block-group scale analysis is aligned with recent calls for equity-focused hazard mapping. Moreover, discrepancies between high flood exposure and moderate DI or SDI values in certain locations highlight the multidimensional nature of vulnerability, challenging assumptions that exposure alone defines risk. This distinction contributes to refined environmental justice diagnostics and supports policy approaches that account for both infrastructural and demographic baselines (Adger 2003; Kryston et al. 2024). Individuals with higher social vulnerability often face systemic barriers to adaptation, including limited financial resources, institutional support, and access to infrastructure upgrades; the multidimensional findings presented here can inform the strategic allocation of public resources, from the expansion of centralized sewerage to targeted buyout programs in persistently at-risk areas.

## Conclusion

This study assessed the vulnerability of OWTS, particularly septic systems, to long-term coastal flooding across Maryland’s Lower Eastern Shore, with the goal of quantifying projected changes in flood exposure and identifying the extent to which these intersect with patterns of social vulnerability. Using hydrodynamic and wave modeling to simulate annual flood occurrence under current and future SLR scenarios, the analysis identified spatial and temporal trends in current and future inundation conditions. These projected flood extents were overlaid with system locations in order to evaluate exposure frequency and identify infrastructure at risk of recurrent inundation. These exposure metrics were also integrated with a social vulnerability index at the Census block-group level, thus providing a spatially resolved framework for understanding how evolving flood hazards may interact with demographic characteristics to shape environmental vulnerability across Maryland’s Chesapeake Bay region.

SLR is projected to substantially increase both the extent and frequency of coastal flooding across Maryland’s Lower Eastern Shore, yet these shifts do not manifest uniformly across the region. Patterns of septic system exposure reflect this heterogeneity, since some counties exhibit gradual accumulation of risk, while others experience more abrupt exposure growth driven by localized low-lying areas and infrastructure distributions. Disparities in social vulnerability further complicate the spatial configuration of projected impacts. While certain inland block groups register elevated values on social vulnerability indices, these areas are not necessarily those with the greatest flood exposure. Moreover, variation between indices alters which communities emerge as comparatively more vulnerable, demonstrating the importance of integrating multiple dimensions of vulnerability rather than relying on a singular metric. Despite substantial SLR-driven increases in flood exposure, only a handful of locations, primarily nearshore Somerset and central Dorchester, are characterized by both high vulnerability and high normalized exposure. Therefore, localized adaptation and infrastructure resilience planning targeting these hotspots may yield more equitable outcomes compared to broad-based regional policies.

While this study provides a spatially detailed assessment of septic system vulnerability under projected SLR, its scope is bounded by several methodological decisions. Flood exposure was evaluated using modeled coastal inundation, without accounting for groundwater rise or compound flooding (i.e., precipitation and riverine flow), both of which may amplify system impacts. In addition, exposure was characterized using flood extent rather than water depth, which may omit localized gradients in inundation severity. For septic systems, however, the primary concern is whether inundation occurs and how frequently it recurs, since repeated flooding is a reasonable proxy for soil saturation and the persistence of waterlogged conditions. Within this regional framework, the use of extent therefore provides a conservative representation of exposure. Nonetheless, future research should incorporate groundwater dynamics and compound flooding processes to better characterize system vulnerability under interacting coastal and hydrologic drivers. Beyond these process-level considerations, uncertainty also persists regarding the pace and magnitude of future SLR and land subsidence, supporting the value of presenting exposure across multiple scenarios. The analysis further evaluates exposure relative to the current distribution of septic systems and does not incorporate projected growth or new installations, thereby isolating changes associated with coastal forcing rather than demographic expansion. Extending this type of assessment to other regions will depend on the availability of parcel-scale infrastructure inventories and technical capacity to implement hydrodynamic modeling workflows. Although many of the environmental datasets required for coastal flood simulation are nationally available, detailed mapping of onsite wastewater systems remains inconsistent across states and local jurisdictions. Applying comparable analyses across additional Mid-Atlantic systems would help evaluate whether the contrast observed between Chesapeake Bay communities and Atlantic-facing areas persists under different geomorphic and tidal conditions. From a decision-making perspective, projected recurrence patterns provide information that can inform revisions to septic siting guidance, long-term permitting decisions for new systems, and prioritization of infrastructure investments. In areas where recurrent flooding coincides with elevated social vulnerability, exposure mapping can also support more strategic targeting of public funding and sewer expansion.

## Supplementary Information

Below is the link to the electronic supplementary material.

Supplementary Material 1

## Data Availability

All data and findings supporting this study are provided in the article and supplementary material. Questions or requests for further information may be directed to the corresponding author.

## Footnotes

1 Conventional septic systems represent one type of OWTS. Other configurations include mound systems, sand filters, and aerobic treatment units, which differ primarily in design complexity and the level or method of wastewater treatment provided.
