# Supplementary material for: Estimating the impacts of recurrent and expanding coastal flooding on septic systems in Maryland’s Chesapeake Bay
Source: Clim Change. 2026 Apr 7;179(4):80. doi: 10.1007/s10584-026-04172-x (PMC13056770; doi:10.1007/s10584-026-04172-x)
Supplement: Supplementary file 1 — Supplementary Material 1 [file 10584_2026_4172_MOESM1_ESM.docx]

# Electronic Supplementary Materials


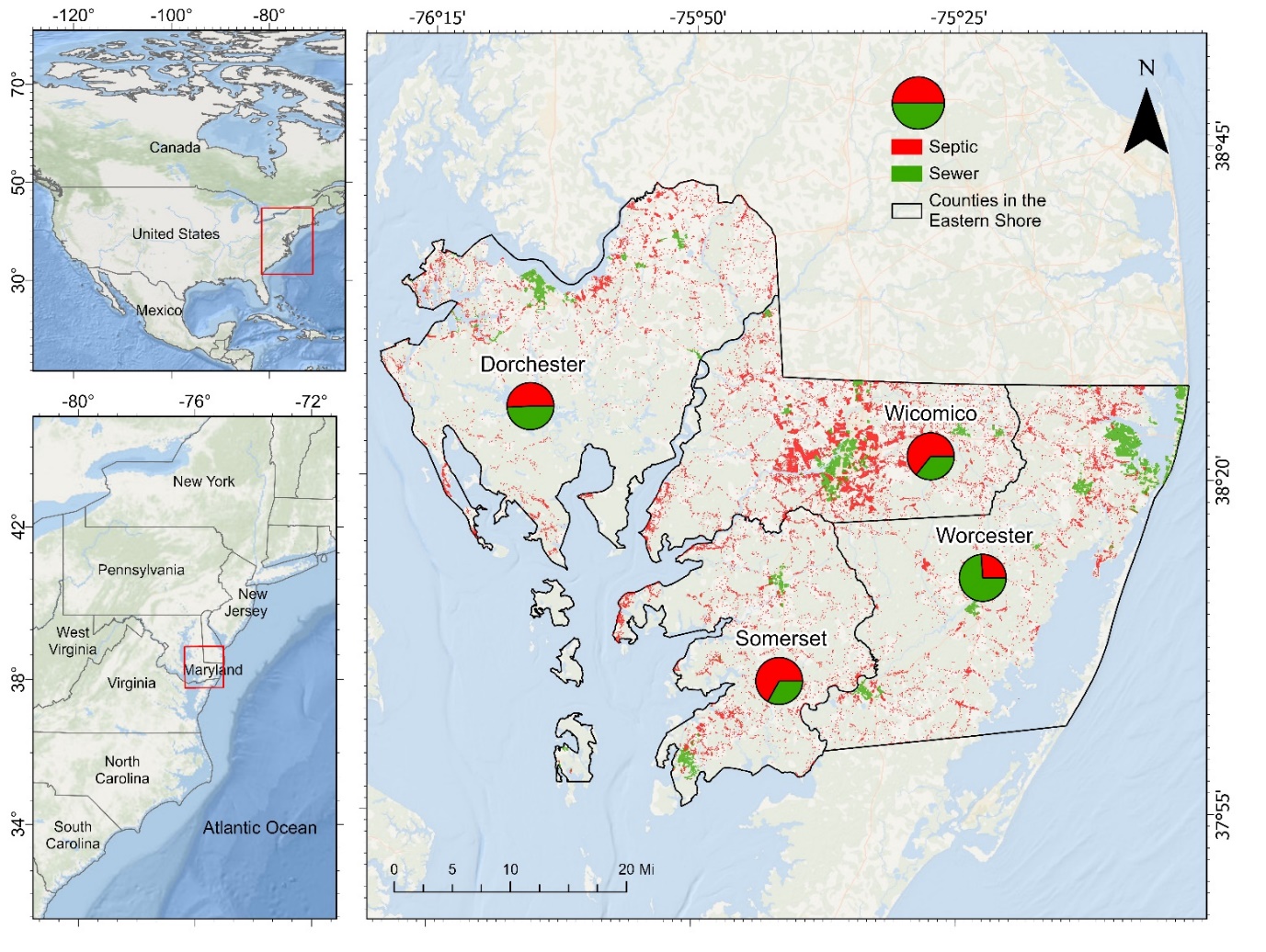


Supplementary Material 1: Distribution of current wastewater infrastructure in Maryland’s Eastern Shore. Points represent locations served by either septic systems or centralized sewer connections.


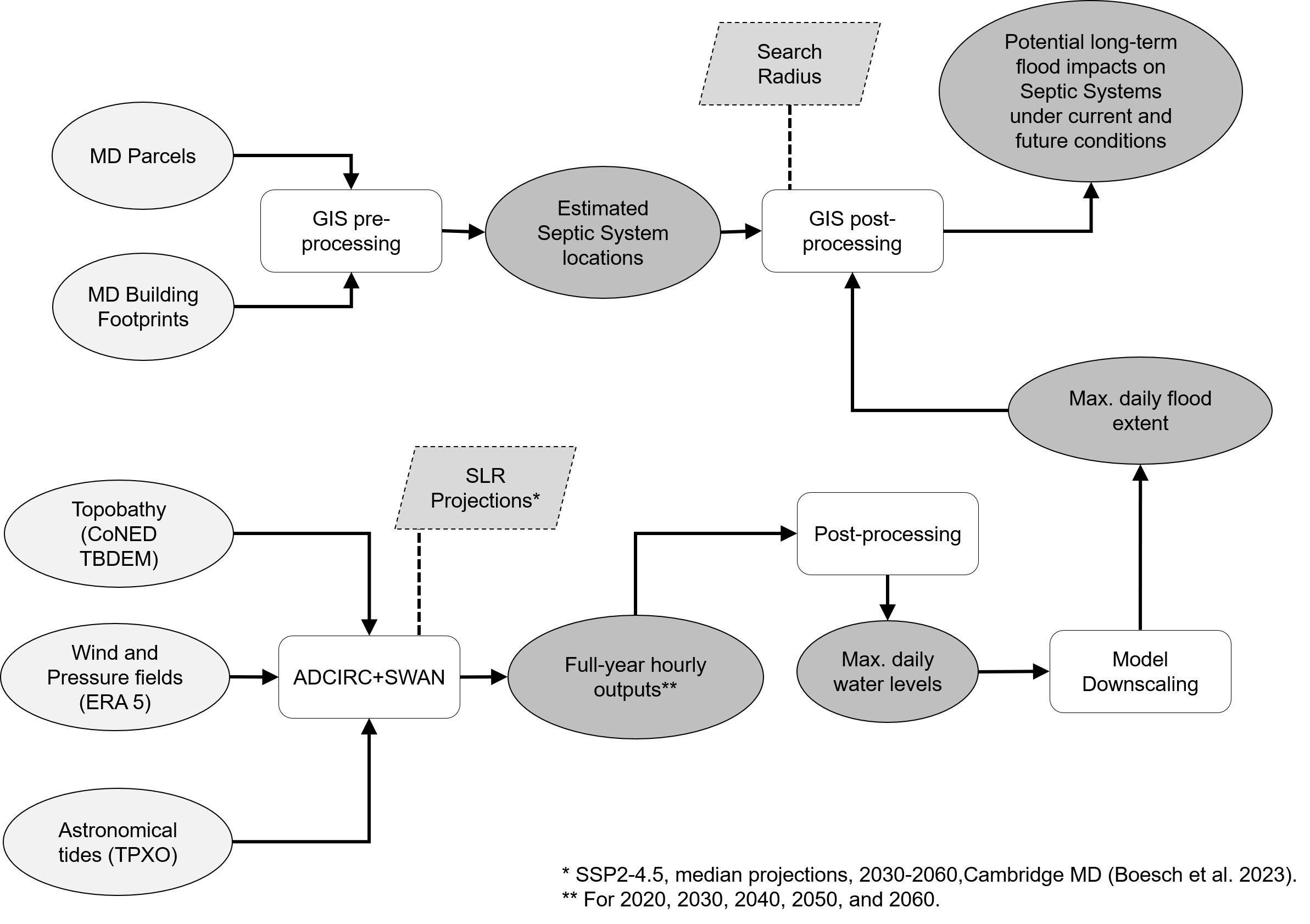


Supplementary Material 2: Numerical modeling and spatial analysis framework for identifying septic system exposure.


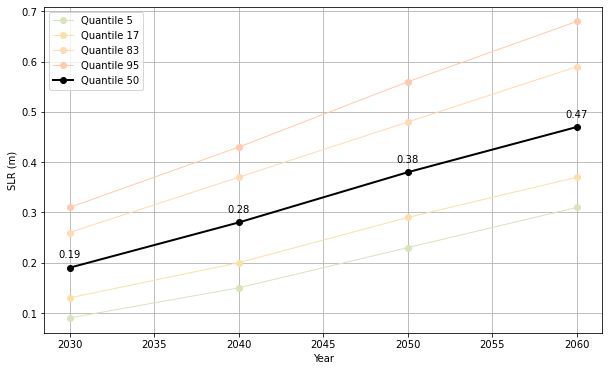


Supplementary Material 3: Sea-level rise projections with quantile probabilities for Cambridge, MD under the SSP2-4.5 emissions scenario. Reported values are cumulative relative to the 2005 baseline year.

Supplementary Material 4: Modeled maximum flood extent (km²) for four counties on Maryland’s Eastern Shore under current (2020) and projected SLR scenarios (2030–2060). Values represent areas expected to flood at least once per year under each scenario, assuming 2020 as baseline hydrodynamic conditions.

|  | **2020** | **2030** | **2040** | **2050** | **2060** |
| --- | --- | --- | --- | --- | --- |
| **Dorchester** | 449.38 | 531.42 | 559.26 | 591.23 | 609.58 |
| **Wicomico** | 45.86 | 56.13 | 58.97 | 63.34 | 65.89 |
| **Somerset** | 197.10 | 234.08 | 247.27 | 261.28 | 269.94 |
| **Worcester** | 66.39 | 84.48 | 95.85 | 98.62 | 103.90 |

Supplementary Material 5: Number of septic systems projected to be exposed to coastal flooding at least once a year under current conditions (2020) and future SLR scenarios (2030–2060). Total septic systems reflect the estimated number of existing systems in the region.

|  | **Total septic systems** | **Impacted in 2020** | **Impacted in 2030** | **Impacted in 2040** | **Impacted in 2050** | **Impacted in 2060** |
| --- | --- | --- | --- | --- | --- | --- |
| **Dorchester** | 11,116 | 731 | 1,230 | 1,421 | 1,619 | 1,709 |
| **Wicomico** | 25,368 | 39 | 59 | 66 | 89 | 95 |
| **Somerset** | 6,543 | 255 | 559 | 762 | 970 | 1,125 |
| **Worcester** | 19,195 | 43 | 183 | 252 | 298 | 346 |

Supplementary Material 6: Number of 1 km² hexagon cells assigned to each dominant flood frequency category in (a) 2020 and (b) 2060, by county. Each hexagon reflects the modal flood recurrence class among septic systems within its extent.

| a) | **Total area (sq. km)** | **Once a year** | **Once every six months** | **Once a month** | **Once a week** | **No flooding** |
| --- | --- | --- | --- | --- | --- | --- |
| **Dorchester** | 2540.55 | 33 | 123 | 0 | 0 | 139 |
| **Wicomico** | 1604.82 | 2 | 11 | 0 | 0 | 23 |
| **Somerset** | 1396.64 | 6 | 45 | 0 | 0 | 122 |
| **Worcester** | 2458.36 | 9 | 0 | 0 | 0 | 53 |

| b) | **Total area (sq. km)** | **Once a year** | **Once every six months** | **Once a month** | **Once a week** | **No flooding** |
| --- | --- | --- | --- | --- | --- | --- |
| **Dorchester** | 2540.55 | 50 | 52 | 142 | 51 | 0 |
| **Wicomico** | 1604.82 | 12 | 6 | 14 | 4 | 0 |
| **Somerset** | 1396.64 | 32 | 52 | 68 | 21 | 0 |
| **Worcester** | 2458.36 | 4 | 25 | 33 | 0 | 0 |


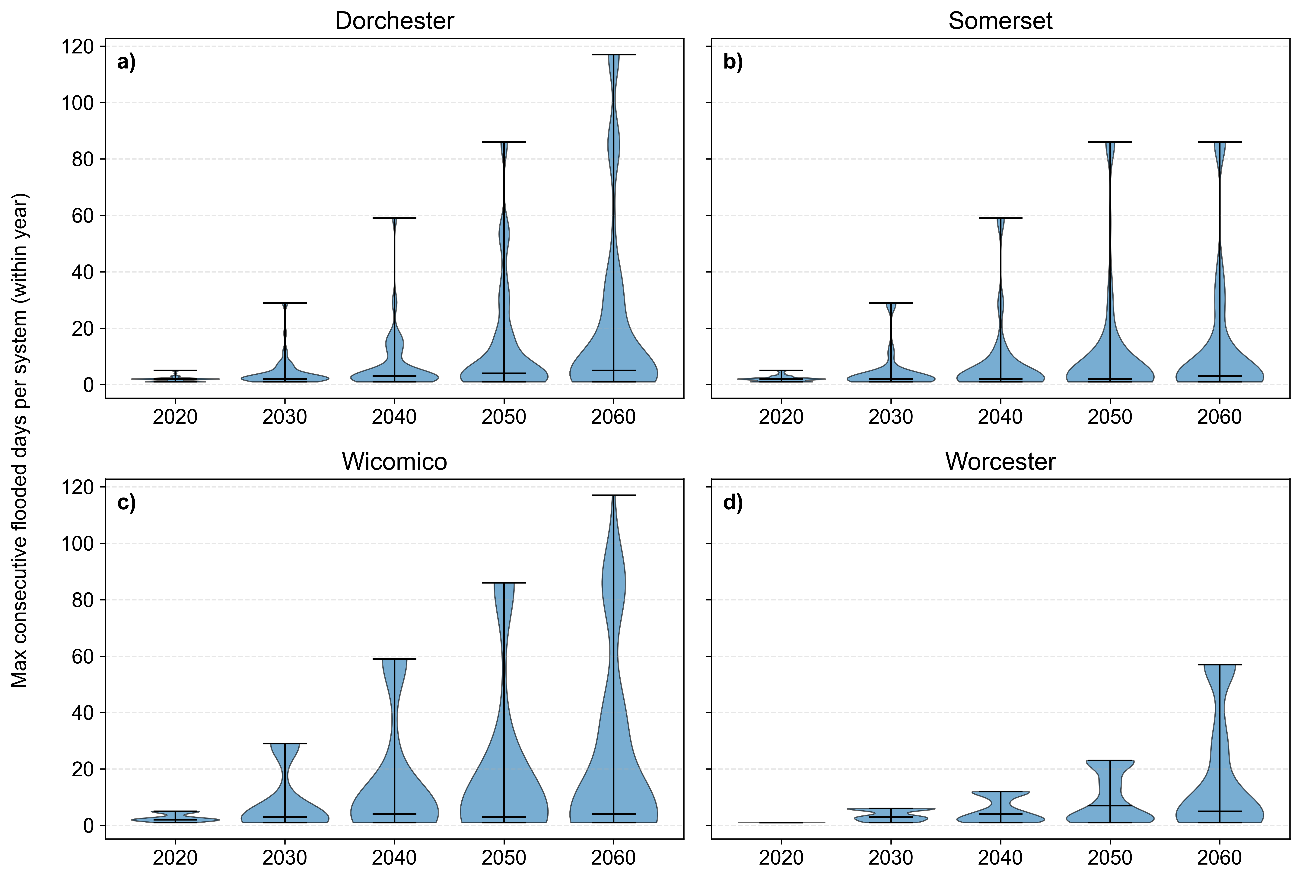


Supplementary Material 7: Distribution of maximum consecutive flooded days per septic system within each simulation year (2020–2060) for (a) Dorchester, (b) Somerset, (c) Wicomico, and (d) Worcester Counties. Each violin represents the distribution of the annual maximum number of back-to-back flooded days per system, computed at daily resolution. The width of each violin reflects the density of systems experiencing a given duration, and the central line indicates the median. Across all counties, the distributions shift toward longer consecutive flooding durations over time, with increasingly pronounced upper tails in later decades, indicating that a growing subset of systems experiences extended, uninterrupted inundation rather than isolated flood days.


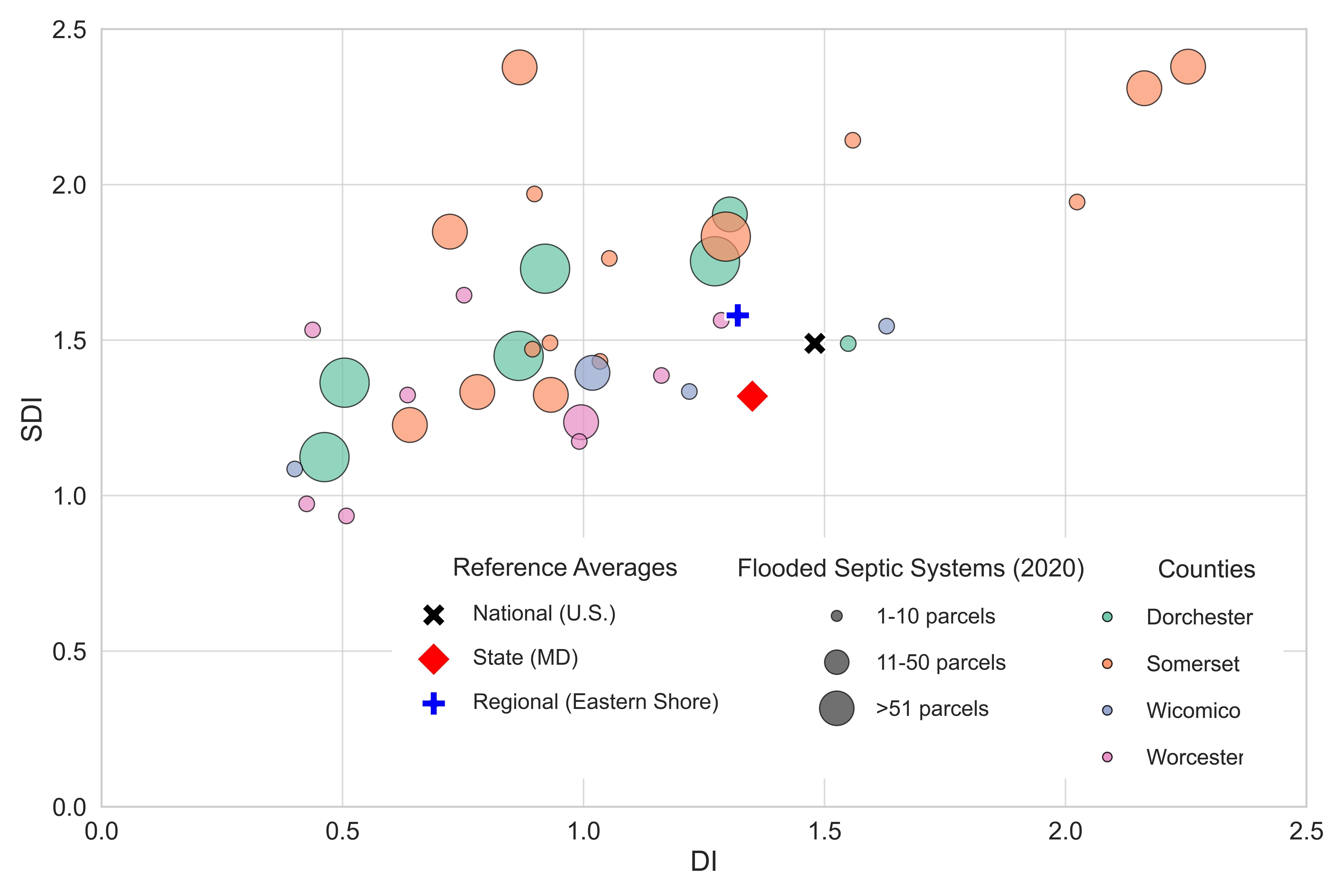


Supplementary Material 8: Demographic Index (DI) and Supplemental Demographic Index (SDI) values for Census block groups with flooded septic systems in 2020. Each point represents a block group; point size corresponds to the number of flooded parcels.


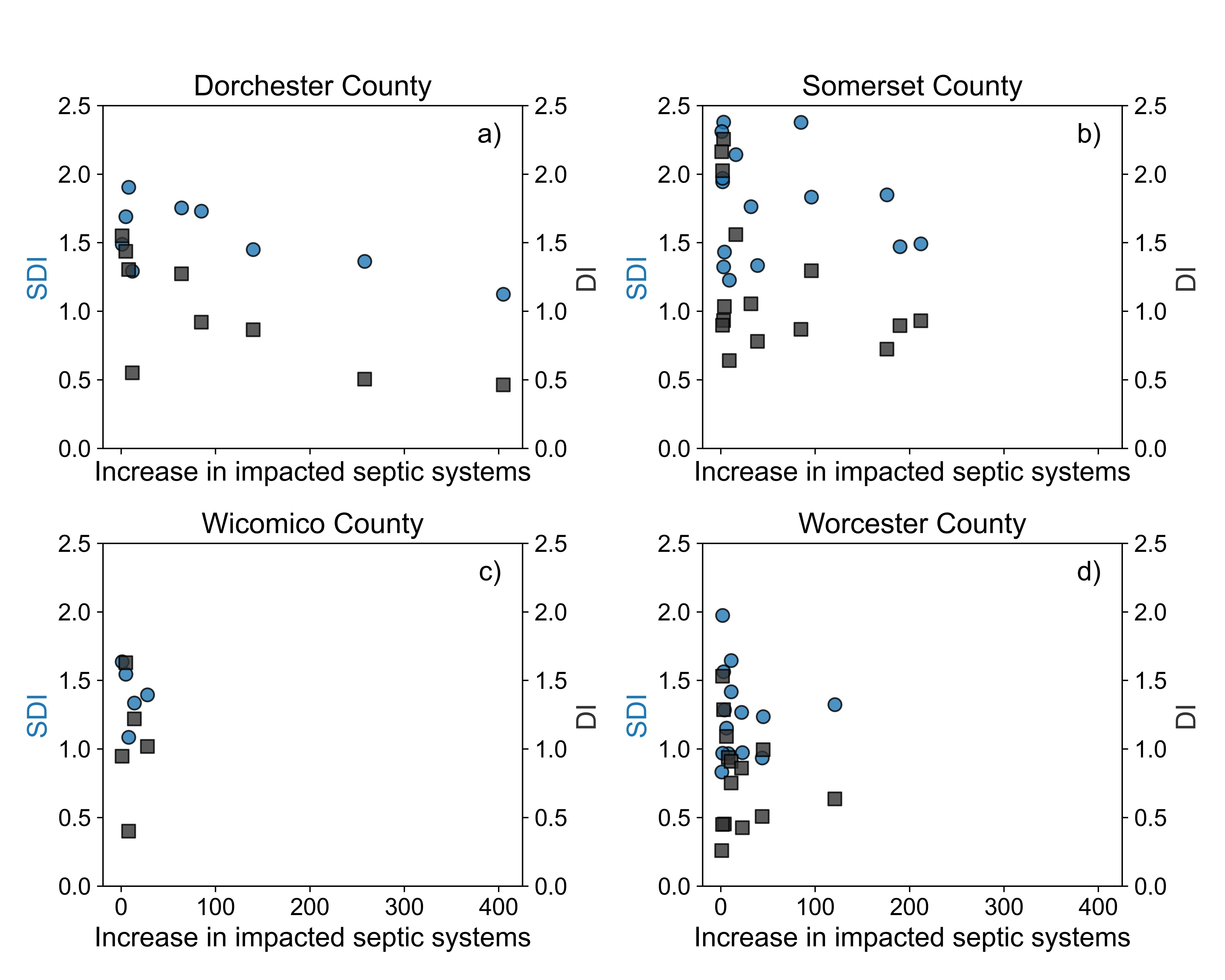


Supplementary Material 9: Projected increase in the number of impacted septic systems from 2020 to 2060 by Census block group, plotted against the SDI (blue circles, left axis) and DI (black squares, right axis). Panels a, b, c and d show results by county. Each point (circle or square) represents a block group with a net increase in the number of affected systems.
